# Intracranial solitary fibrous tumor mimicking meningioma: A case report

**DOI:** 10.1097/MD.0000000000023504

**Published:** 2020-12-11

**Authors:** Longyang Cheng, Hongbin Ni, Yuxiang Dai

**Affiliations:** Department of Neurosurgery, Drum Tower Hospital Affiliated to the Medical School of Nanjing University, Nanjing, Jiangsu Province, China.

**Keywords:** clinical manifestation, diagnosis, solitary fibrous tumor, treatment

## Abstract

**Rationale:**

Solitary fibrous tumor is a rare mesenchymal tumor. This case report describes the diagnosis and treatment of this tumor.

**Patient concerns:**

A 31-year-old patient presented with epileptic seizure and headache 1 day prior to the visit and showed transient right limb hemiplegia for 6 hours that was resolved after intravenous infusion of mannitol.

**Diagnoses:**

Based on imaging, the provisional diagnosis was meningioma. Postsurgical histopathological diagnosis confirmed solitary fibrous tumor.

**Interventions:**

The lesion was totally excised. The patient improved remarkably after the operation, without any signs of associated limb movement disorder. No epileptic seizure was observed or reported after the operation.

**Outcomes:**

Postoperation computed tomography (CT) scans showed no obvious residual tumor. The patient was followed up every 3 months for a total of 1 year following the operation, during which time the patient did not complain of headache or seizure.

**Lessons:**

The manifestation of solitary fibrous tumor (SFT) through imaging methods has certain specific findings,butimmunohistochemistry is still very important for confirming the diagnosis.

## 1 Introduction

Solitary fibrous tumor (SFT), a rare mesenchymal tumor, was first described as a spindle-cell tumor of the pleura by Klemperer and Rabin in 1931.^[1]^ Although SFT most often arises in the pleural cavity, it has also been described in a number of locations outside of the thorax, including the orbit, nasal cavities, paranasal sinuses, thyroid glands, parotid glands, buccal cavity, and the central nervous system (CNS). Before 1996, intracranial SFT (ISFT) was diagnosed as either fibroblastic meningioma or hemangiopericytoma.^[2,3]^ Carneiro et al^[4]^ first described 7 cases of meningeal SFT and showed that they could be morphologically and immunohistochemically distinguished from fibroblastic meningioma. Due to its rarity and resemblance to meningioma and hemangiopericytomas, intracranial SFT has been confused with other types of brain tumors. ISFT is often poorly recognized and remains a diagnostic challenge. Our case report showed several features that indicated a diagnosis of meningioma, but postsurgical histopathology confirmed the diagnosis of SFT.

## 2 Case presentation

**Figure 1:**
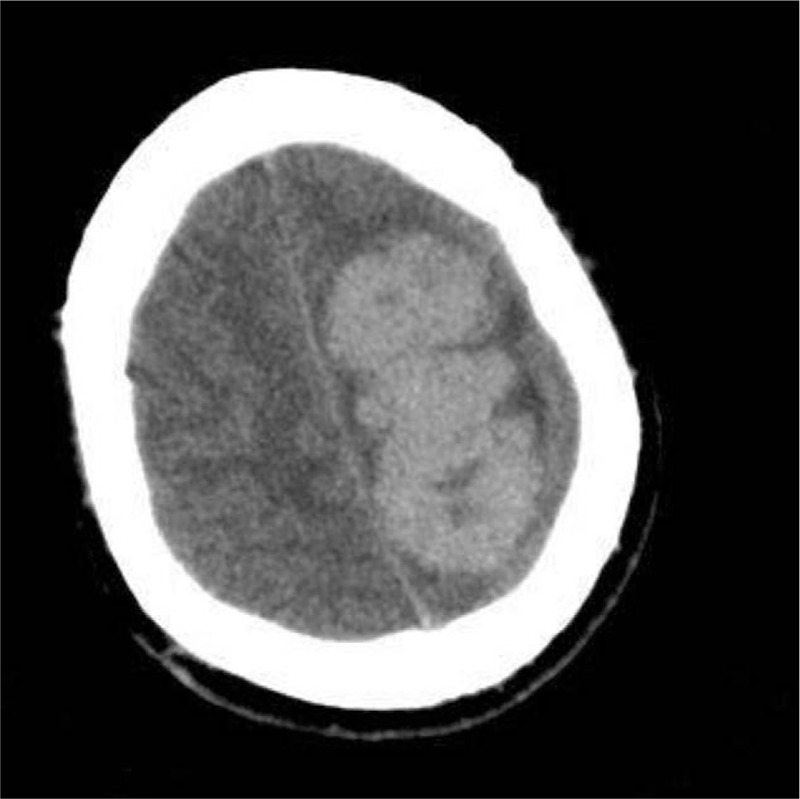
CT scans showed a high-density lesion in the left frontoparietal lobe. CT = computed tomography.

**Figure 2:**
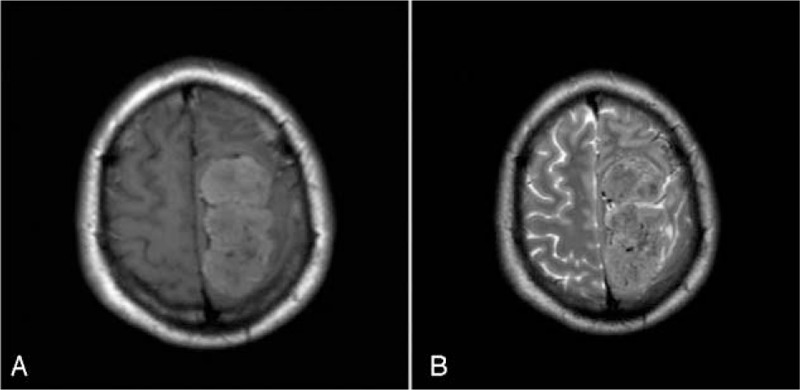
Brain MRI revealed (A) high mixed-intensity signals on the T1-weighted MRI, and (B) high mixed-intensity signals on the T2-weighted MRI. MRI = magnetic resonance imaging.

**Figure 3:**
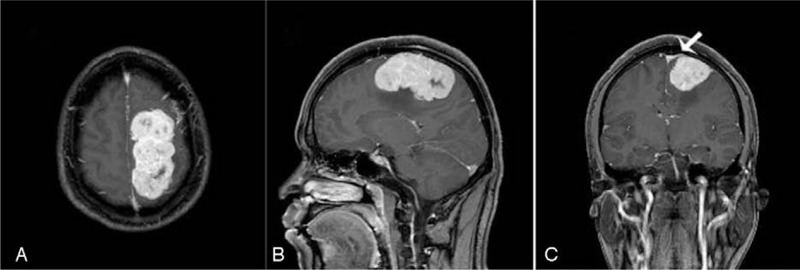
MRI revealed strong contrast enhancement on (A) the horizontal plane, (B) sagittal plane, and (C) frontal plane; “dural tail” sign can be seen in the frontal plane. MRI = magnetic resonance imaging.

A 31-year-old male patient presented with the first epileptic seizure and headache for 1 day prior to the visit. The patient also showed transient right limb hemiplegia for 6 hours that was resolved after intravenous infusion of mannitol. A non-contrast brain computed tomography (CT) scan showed a large 7.5 × 3.6 × 4 cm mass, and high-density lesions in the left frontoparietal lobe, and across central sulcus with precentral gyrus compression (Fig. 1). A brain magnetic resonance imaging (MRI) revealed high mixed-intensity signals on a T1-weighted MRI (Fig. 2A), high mixed-intensity signals on a T2-weighted MRI (Fig. 2B), and strong contrast enhancement with dural tail signs (Fig. 3). According to the patient's clinical history and radiological examination results, he was diagnosed as meningioma. Operative findings confirmed that the mass had widely invaded the meninges. The lesion was totally removed, and a diagnosis of meningioma was suspected. The excised specimen was sent for pathology. The patient improved remarkably after the operation, without any signs of associated limb movement disorder. The patient suffered from intermittent headaches for 1 week after the operation. After intravenous administration of mannitol and the intravenous injection of analgesics, the patient's symptoms were relieved. No epileptic seizure was observed or reported after the operation. We followed up on the patient every 3 months for a total of 1 year following the operation, during which time the patient did not complain of a headache or experience an epileptic seizure. Postoperation CT scans showed no obvious residual tumor.

**Figure 4:**
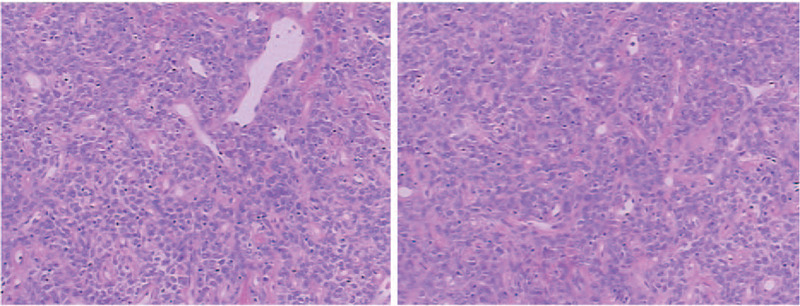
Microscopic examination showed thin-walled vessels, short spindle cells, and oval cell hyperplasia, but no increase in mitotic activity.

**Figure 5:**
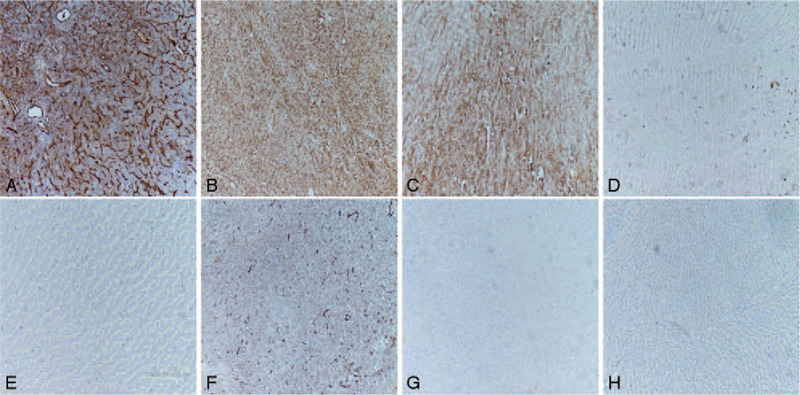
Immunohistochemical staining. Representative images of (A) CD34, (B) Bcl-2, (C) CD99, (D) Ki-67, (E) EMA, (F) SMA, (G) PR, (H) GFAP. Showed that the tumor cells were diffusely positive for CD34, Bcl-2, CD99, but negative for EMA, PR, and GFAP, moderate staining for SMA with the proliferation labeling index of Ki-67 <2%. Bcl-2 = B-cell lymphoma-2, CD34 = hematopoietic progenitor cell antigen, CD99 = cluster of differentiation 99, EMA = epithelial membrane antigen, GFAP = glial fibrillary acidic protein, Ki-67 = Ki67 antibody, PR = progesterone receptor, SMA = moderate staining for smooth muscle actin.

Microscopic examination showed thin-walled vessels, short spindle cells and oval cell hyperplasia, but no increase in mitotic activity (Fig. 4). Immunohistochemical staining showed that the tumor cells were diffusely positive for hematopoietic progenitor cell antigen (CD34), B-cell lymphoma (Bcl-2), cluster of differentiation 99, but negative forepithelial membrane antigen (EMA), progesterone receptor (PR), and glial fibrillary acidic protein (GFAP), moderate staining for smooth muscle actin (SMA) with the proliferation labeling index of Ki67 antibody <2% (Fig. 5). The immunohistochemical profile in our case was strongly suggestive of SFT.

## 3 Discussion

SFT is a rare spindle-cell neoplasm of mesenchymal origin that most frequently arises in the pleural cavity, but recently it has been found to occur in other sites. Extra-pleural SFTs can mimic other benign or malignant spindle cell tumors, rendering clinical and histological diagnoses difficult. The fact that SFTs can involve the CNS has been established since 1996.^[4]^ Since then, increasing numbers of ISFT cases of CNS have been reported. A differential diagnosis should include meningioma, hemangiopericytoma, fibrosarcoma, and schwannoma. Meningioma and hemangiopericytoma are especially important for the differential diagnosis.

ISFTs occur most commonly in the posterior cranial fossa, without any noted sex differences. Clinical symptoms are related to the tumor location. Tumors that occur in the cerebral hemisphere often cause headache, nausea, vomiting, seizure, and other symptoms, while hearing loss, unsteady gait, and brainstem compression symptoms occur when the tumor is in the cerebellopontine angle (CPA) area.^[5,6]^ The primary presenting symptom in this case was a headache in patient. The other presenting symptom was an epileptic seizure, because of the occurrence of the tumor in parietal convexity.

Most ISFTs are still radiologically misdiagnosed and mistaken for meningiomas or schwannomas. A brain CT scan shows a clear edge and isolated mass. Density of the mass is generally uniform, while necrotic area may be low in density. On MRI scans, SFT is usually isointense with normal cerebral parenchyma on T1-weighted images, and iso- to hypointense on T2-weighted images and shows homogeneous enhancement after the intravenous administration of the contrast agent gadopentetate dimeglumine.^[7,8]^ Moreover, the typical “dural tail” sign classically seen in meningiomas is not usually present. It is of interest to note that the MRI in this case revealed hyperintense SFT on the T1-weighted MRI (Fig. 2A) and T2-weighted MRI (Fig. 2B), along with strong enhancement with dural tail signs (Fig. 3A–C), all of which led to our preoperative diagnosis of meningioma.

Microscopically, the histological features of ISFT are similar to the SFT in other parts. It is mainly composed of spindle shaped cells. These cells tend to be bundled in barely undulating fascicles and lack any specific arrangement, and thus often result in a “patternless pattern.” Deposition of collagen substance is increased in the cell sparse area. Crack or staghorn-like vascular is often prominent in the cell-intensive areas, characterized by small and/or large branching vascular spaces. With the development of immunohistochemistry, it is thought that immunohistochemical examinations are now essential for differentiating SFTs. SFT shows a diffuse and strong positivity for CD34 in 80% to 100% of cases, frequent positive reactions for Bcl-2 and vimentin, but negative reactions for EMA and S-100.^[9]^ However, this is not an absolute differentiating point, as hemangiopericytoma can also be positive for CD34 and Bcl-2, though these can be partially and weakly positive compared with SFT. Additionally, in the differentiation of meningioma, fibrous meningioma often shows characteristic psammoma bodies, which are absent in SFTs. Besides, compared with SFT, a fibrous meningioma is usually better stained by an EMA antibody, but only mildly or focally positive on CD34 and Bcl-2 staining. The CD34 staining for SFT is usually strong and diffuse but is usually negative for the EMA, SMA, PR, and GFAP.^[10–13]^ In this article, we observed moderate staining for SMA and the reason may be related to tissue extraction and staining. The immunohistochemical profile in our case was strongly suggestive of SFT.

The treatment of choice is surgical resection, with supplemental radiotherapy and chemotherapy if necessary. The surgical strategy depends on the location and size of the tumor. Radical resection is recommended in order to prevent tumor recurrence and metastasis. Local recurrences have been reported with incomplete surgical excision.^[14]^ Some cases with malignant potential have also been reported, including metastases.^[15]^ We cannot determine the prognosis based solely on the morphology of SFT. Some cases with seemingly benign morphology may often relapse or metastasize. Alternatively, some patients can have tumors that are biologically benign even though the morphology may appear malignant.

ISFTs are much less common than meningiomas. The ISFT in this case had one unusual feature which was the radiological appearance of a “dural tail” mimicking meningioma. This case report highlights the fact that the typical radiological appearance of one lesion may sometimes be misleading. Knowledge of these tumors is essential for neurosurgeons to include them in the preoperative differential diagnosis.

## Author contributions

Yuxiang Dai and Hongbin Ni was responsible for the operation and specimen collection. Longyang Cheng read literature, collected data and wrote manuscript.
